# Supplementary figures and images for: Association between serum endocan levels and organ failure in hospitalized patients with cirrhosis
Source: PLoS One. 2024 Dec 26;19(12):e0315619. doi: 10.1371/journal.pone.0315619 (PMC11671009; doi:10.1371/journal.pone.0315619)

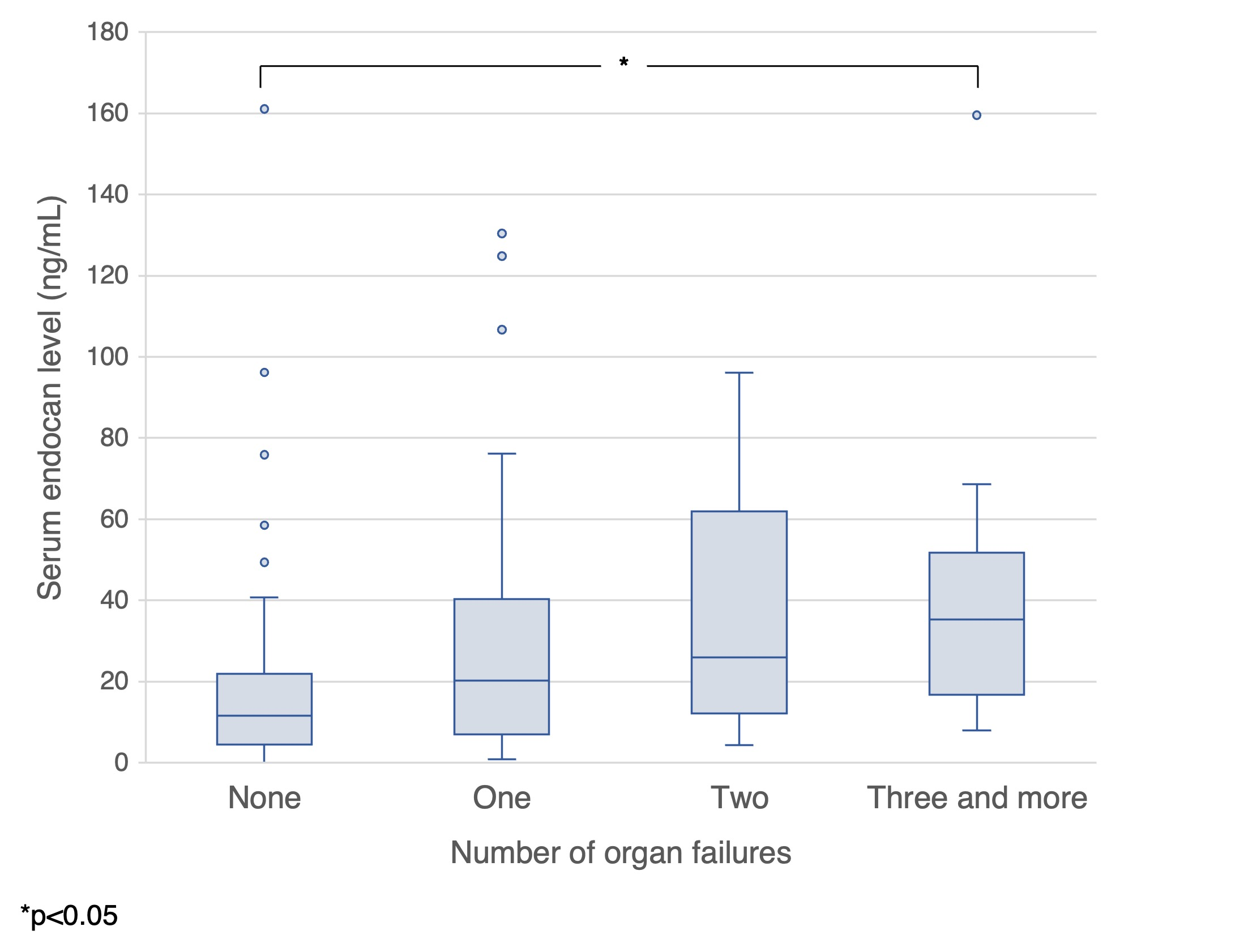

Supplement: S1 Fig — The level of serum endocan increased as the number of organ failures increased. (TIF) [file pone.0315619.s001.tif]

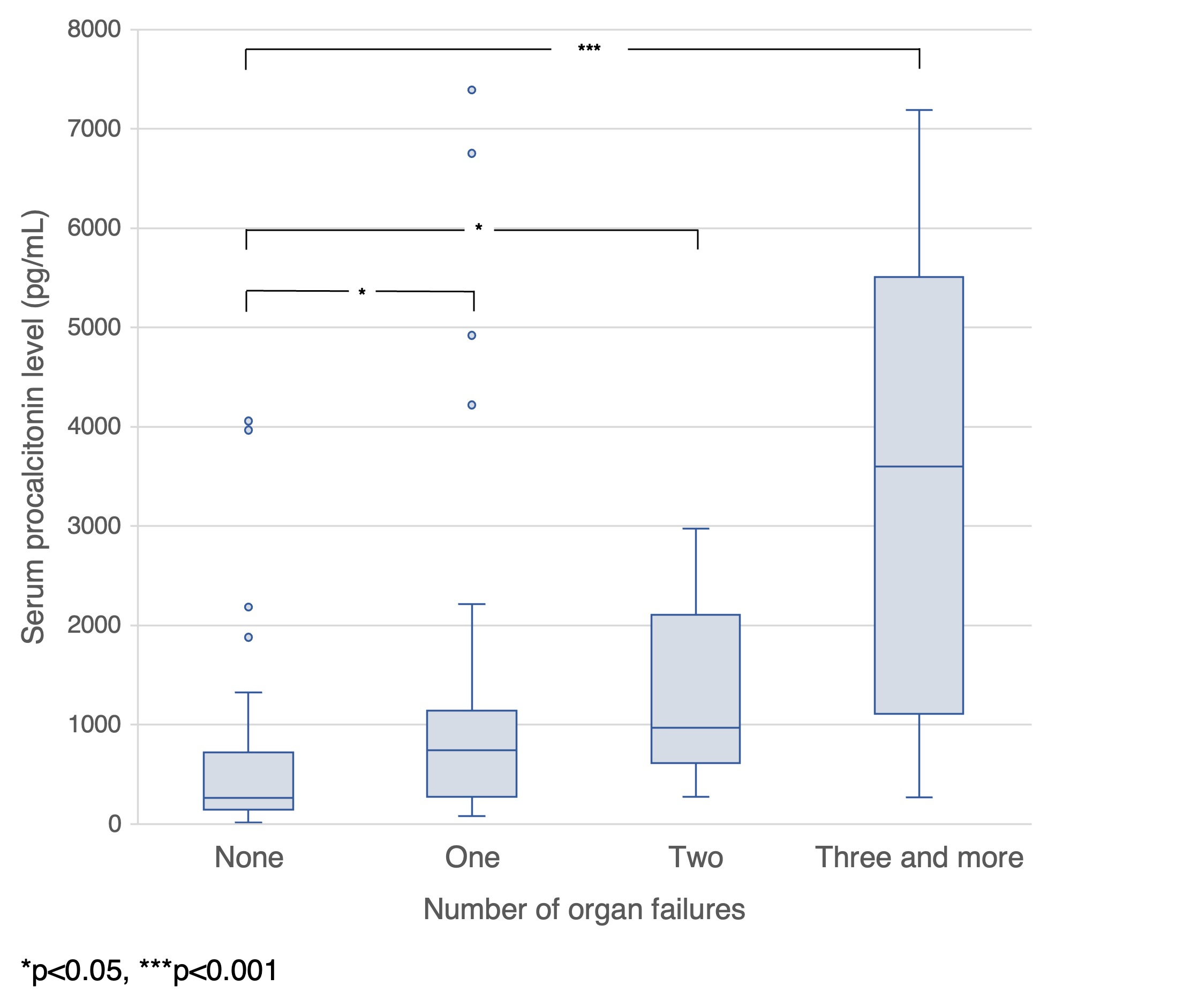

Supplement: S2 Fig — The level of serum procalcitonin increased as the number of organ failures increased. (TIF) [file pone.0315619.s002.tif]

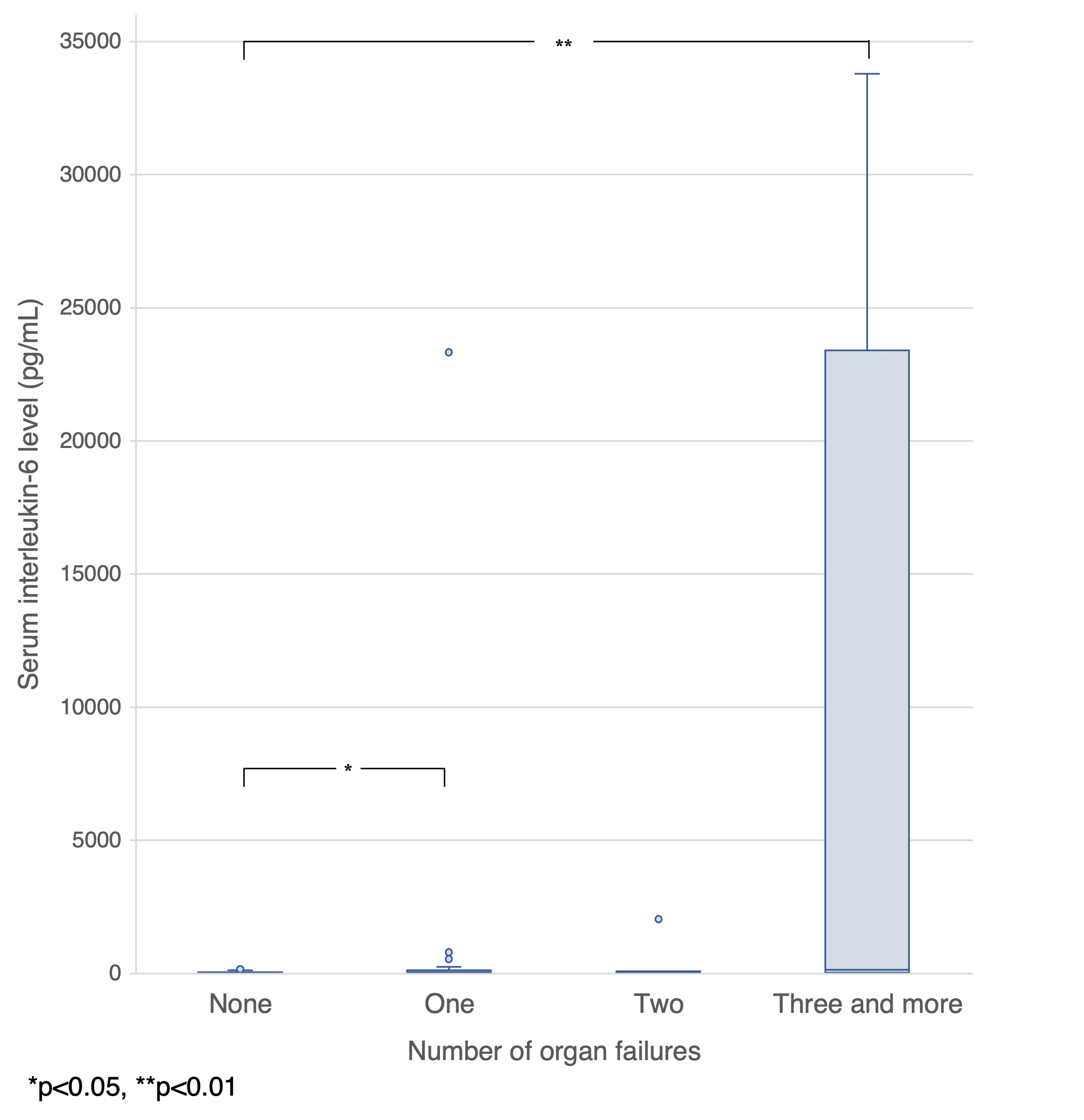

Supplement: S3 Fig — The level of serum interleukin-6 increased as the number of organ failures increased. (TIF) [file pone.0315619.s003.tif]
