# Supplementary material for: Association between serum endocan levels and organ failure in hospitalized patients with cirrhosis
Source: PLoS One. 2024 Dec 26;19(12):e0315619. doi: 10.1371/journal.pone.0315619 (PMC11671009; doi:10.1371/journal.pone.0315619)
Supplement: S1 Table — (DOCX) [file pone.0315619.s004.docx]

**S1 Table. Level of serum endocan according to type of organ failure.**

|  | **Serum endocan (ng/mL)** | | | | |
| --- | --- | --- | --- | --- | --- |
| **OF** | **No OF** | | **OF** | | **p-value** |
|  | **n** | **Median (IQR)** | **n** | **Median (IQR)** |  |
| Liver | 87 | 12.19 (6.08-34.26) | 29 | 25.90 (9.30-57.68) | 0.047 |
| Kidney | 96 | 13.31 (6.78-34.32) | 20 | 15.99 (7.20-48.00) | 0.737 |
| Cerebral | 99 | 12.03 (5.92-34.26) | 17 | 29.68 (18.73-47.46) | 0.004 |
| Coagulation | 101 | 12.12 (6.31-32.40) | 15 | 33.36 (17.05-53.45) | 0.007 |
| Cardiovascular | 108 | 12.70 (6.62-33.32) | 8 | 42.11 (23.89-90.23) | 0.005 |
| Respiratory | 113 | 13.41 (6.92-34.89) | 3 | 16.14 (12.19-) | 0.584 |

IQR, interquartile range; OF, organ failure
