# Supplementary material for: Association between serum endocan levels and organ failure in hospitalized patients with cirrhosis
Source: PLoS One. 2024 Dec 26;19(12):e0315619. doi: 10.1371/journal.pone.0315619 (PMC11671009; doi:10.1371/journal.pone.0315619)
Supplement: S2 Table — (DOCX) [file pone.0315619.s005.docx]

**S2 Table. Level of serum procalcitonin according to type of organ failure.**

|  | **Serum procalcitonin (pg/mL)** | | | | |
| --- | --- | --- | --- | --- | --- |
| **OF** | **No OF** | | **OF** | | **p-value** |
|  | **n** | **Median (IQR)** | **n** | **Median (IQR)** |  |
| Liver | 87 | 301.12  (164.53-872.31) | 29 | 1298.59  (703.88-3,597.81) | <0.001 |
| Kidney | 96 | 342.31  (170.55-1,075.83) | 20 | 904.94  (485.96-3,347.19) | 0.007 |
| Cerebral | 99 | 456.44  (190.28-1,078.74) | 17 | 551.29  (233.69-3,597.81) | 0.343 |
| Coagulation | 101 | 346.37  (177.77-983.60) | 15 | 1860.84  (565.26-4,919.27) | 0.001 |
| Cardiovascular | 108 | 427.74  (186.42-1,052.10) | 8 | 5563.88  (987.46-23,663.33) | 0.002 |
| Respiratory | 113 | 456.44  (191.66-1,270.18) | 3 | 615.08  (551.29-) | 0.389 |

IQR, interquartile range; OF, organ failure
