# Supplementary material for: Association between serum endocan levels and organ failure in hospitalized patients with cirrhosis
Source: PLoS One. 2024 Dec 26;19(12):e0315619. doi: 10.1371/journal.pone.0315619 (PMC11671009; doi:10.1371/journal.pone.0315619)
Supplement: S3 Table — (DOCX) [file pone.0315619.s006.docx]

**S3 Table. Level of serum interleukin-6 according to system of organ failure.**

|  | **Serum interleukin-6 (pg/mL)** | | | | |
| --- | --- | --- | --- | --- | --- |
| **OF** | **OF** | | **No OF** | | **p-value** |
|  | **n** | **Median (IQR)** | **n** | **Median (IQR)** |  |
| Liver | 87 | 28.67 (12.34-76.40) | 29 | 52.40 (31.59-153.64) | 0.017 |
| Kidney | 96 | 34.61 (12.97-73.22) | 20 | 65.35 (29.05-121.26) | 0.052 |
| Cerebral | 99 | 30.17 (12.97-73.85) | 17 | 67.71 (37.37-173.87) | 0.011 |
| Coagulation | 101 | 29.78 (12.77-73.85) | 15 | 67.71 (38.89-247.28) | 0.003 |
| Cardiovascular | 108 | 34.61 (12.99-72.00) | 8 | 11054.54 (113.65-32566.53) | <0.001 |
| Respiratory | 113 | 37.05 (13.28-86.48) | 3 | 37.68 (29.14-) | 0.774 |

IQR, interquartile range; OF, organ failure
