# Supplementary material for: Association between serum endocan levels and organ failure in hospitalized patients with cirrhosis
Source: PLoS One. 2024 Dec 26;19(12):e0315619. doi: 10.1371/journal.pone.0315619 (PMC11671009; doi:10.1371/journal.pone.0315619)
Supplement: S4 Table — (DOCX) [file pone.0315619.s007.docx]

**S4 Table.** **Patient characteristics and baseline laboratory parameters according to 28-day mortality** (n = 112).

| **Variables** | **Survivors (n = 90)** | **Non-survivors (n = 22)** | ***p*-value** |
| --- | --- | --- | --- |
| Age (years), mean ±S.D. | 60 ± 17 | 60 ± 14 | 0.20 |
| Male sex, n (%) | 52 (57.8) | 16 (72.7) | 0.94 |
| **Etiology of cirrhosis, n (%)** | | | |
| Alcohol | 27 (30) | 7 (31.8) | 0.87 |
| HBV | 19 (21.1) | 8 (36.4) | 0.13 |
| MASLD | 15 (16.7) | - | 0.04 |
| Cryptogenic | 12 (13.3) | 3 (13.6) | 1.00 |
| Other | 9 (10) | 3 (13.6) | - |
| HCV | 8 (8.9) | 1 (4.6) | 0.69 |
| **Comorbidity, n (%)** | | | |
| Diabetes mellitus | 35 (38.9) | 4 (18.2) | 0.07 |
| Hypertension | 29 (32.2) | 5 (22.7) | 0.39 |
| Cardiovascular disease | 9 (10) | - | 0.20 |
| Chronic kidney disease | 8 (8.9) | 1 (4.5) | 0.69 |
| No comorbidity | 25 (27.8) | 12 (54.5) | 0.02 |
| **Indication of admission, n (%)** | | | |
| Bacterial infection | 32 (35.6) | 5 (22.7) | 0.03 |
| GI bleeding | 29 (32.2) | 7 (31.8) | 0.97 |
| **Laboratory baseline (median, IQR)** | | | |
| NLR | 5.30 (3.18-8.97) | 7.83 (3.68-21.93) | 0.04 |
| Platelet (10^3^/µL) | 121 (75-185) | 107 (67-164) | 0.34 |
| INR | 1.45 (1.26-1.68) | 2.56 (1.81-4.47) | <0.001 |
| Creatinine (mg/dL) | 1.00 (0.82-1.51) | 1.13 (0.78-2.14) | 0.35 |
| Sodium (mmol/L) | 135 (130-138) | 131 (127-134) | 0.04 |
| TB (mg/dL) | 2.11 (0.96-5.84) | 16.03 (6.00-28.55) | <0.001 |
| Albumin (g/dL) | 2.9 (2.4-3.4) | 2.4 (2.1-2.8) | 0.01 |
| Lactate (mmol/L) | 2.7 (1.5-4.8) | 3.4 (2.6-4.5) | 0.15 |
| **Biomarker (median, IQR)** | | | |
| Endocan (ng/mL) | 12.24 (6.95-34.26) | 28.22 (15.10-57.24) | 0.02 |
| PCT (pg/mL) | 302.17 (167.21-945.09) | 1466.44 (561.77-4454.93) | <0.001 |
| IL-6 (pg/mL) | 26.83 (11.40-60.50) | 84.16 (51.26-397.29) | <0.001 |
| **Severity score** | | | |
| CTP score | 9 (7-10) | 12.5 (11.75-13) | <0.001 |
| CTP grade, n (%) |  |  |  |
| A | 18 (20) | - | 0.02 |
| B | 39 (43.3) | 4 (18.2) | 0.03 |
| C | 33 (36.7) | 18 (81.8) | <0.001 |
| MELD score | 15 (11-22) | 31 (22-36) | <0.001 |
| CLIF-ACLF score | 46 (43.5-52.5) | 57.5 (54-69.75) | <0.001 |
| AARC score | 8 (7-10) | 11 (10-13) | <0.001 |
| **OF, n (%)** | 34 (37.8) | 19 (86.4) | <0.001 |
| Liver | 15 (16.7) | 13 (59.1) | <0.001 |
| Kidney | 13 (14.4) | 6 (27.3) | 0.20 |
| Cerebral | 9 (10) | 8 (36.4) | 0.01 |
| Coagulation | 4 (4.4) | 11 (50) | 0.001 |
| Cardiovascular system | 4 (4.4) | 4 (18.2) | 0.046 |
| Respiratory system | 1 (1.1) | 2 (9.1) | 0.01 |
| New OF in admission, n (%) | 2 (2.2) | 11 (50) | <0.001 |
| Hospital-acquired infection, n (%) | 12 (13.3) | 10 (45.5) | 0.002 |

AARC, Asian Pacific Association for the Study of the Liver (APASL) ACLF Research Consortium; CLIF-C ACLF, Chronic Liver Failure Consortium acute-on-chronic liver failure; CTP, Child-Turcotte-Pugh; GI, gastrointestinal; HBV, Hepatitis B virus; HCV, Hepatitis C virus; IL-6, interleukin-6; INR, international normalized ratio; IQR, interquartile range; MASLD, metabolic dysfunction-associated steatotic liver disease; MELD, model for end-stage liver disease; NLR, neutrophil-to-lymphocyte ratio; OF, organ failure; PCT, procalcitonin; TB, total bilirubin
