# Supplementary material for: Association between serum endocan levels and organ failure in hospitalized patients with cirrhosis
Source: PLoS One. 2024 Dec 26;19(12):e0315619. doi: 10.1371/journal.pone.0315619 (PMC11671009; doi:10.1371/journal.pone.0315619)
Supplement: Attachment — Submitted filename: Response to Reviewers PLOS One endocan.docx [file pone.0315619.s008.docx]

**Kessarin Thanapirom, M.D., PhD.**
Division of Gastroenterology

Department of Medicine, Faculty of Medicine

Chulalongkorn University, Bangkok, Thailand

Phone: +662 256 4265

Email: [Kessarin.t@chula.ac.th](mailto:Kessarin.t@chula.ac.th)

November 5^th^, 2024

Dear Editor, ***PLOS ONE***

Thank you for considering our manuscript entitled, **“Association between serum endocan levels and organ failure in hospitalized patients with cirrhosis”** by Wejnaruemarn, et al to be published in ***PLOS ONE*** (Manuscript number PONE-D-24-36598).

We are deeply grateful to all of the reviewers for their assistance in enhancing the quality of our work. We have thoroughly evaluated the comments. Moreover, the manuscript has been carefully reviewed by an experienced editor whose first language is English. The certificate is available for your review. The modifications are indicated in yellow within the main manuscript. The following are the point-by-point responses to the comments of reviewers and editors.

**Reviewer 1**

The study titled "Association of serum endocan with organ failure in hospitalized cirrhotic patients" is well-written with appropriate methodology and interesting findings. I have some comments for improvement:

**Comment 1**

Define abbreviations in their first use and make sure that abbreviated forms are being used after the definition

**Response 1**

Thank you for your remarks. We have carefully reviewed the manuscript and made the necessary correction as per your recommendations.

**Revised in the methods section:**

“Asian Pacific Association for the Study of the Liver ACLF Research Consortium (AARC) score” (Page 6, line 97).

**Comment 2**

Add the strengths of your study before the limitations section.

**Response 2**

Thank you for your valuable comment. We have incorporated the strengths prior to the limitation section.

**Revised in the discussion section:**

“The study’s main strength lies in validating the diagnostic value of serum endocan, along with widely used biomarkers, such as PCT and IL-6, for detecting OF in hospitalized patients with cirrhosis. The accuracy of these biomarkers was comparable, demonstrating their potential utility in providing physicians with an additional reliable method for identifying OFs in this population. Another notable strength of this study was that serum endocan served as an independent factor for OF and, when combined with other independent variables, produced a high AUC for detecting OF. However,..” (Page 21, line 322-328).

**Comment 3**

Use data from previous meta-analyses investigating the role of endocan in other diseases including COVID-19, ARDS, hypertension, OSA, diabetes, CKD, etc.

**Response 3**

We appreciate your insightful comment. We have ensured that the data from previous studies are included in the manuscript.

**Revised in the introduction section:**

“Previous meta-analyses have shown that high serum endocan levels are associated with various diseases, including hypertension, diabetes mellitus, chronic kidney disease, and acute respiratory distress syndrome (ARDS) [10-13]” (Page 5, line 70-72).

**Revised in the discussion section:**

“In addition to OF in sepsis, another area of extensive research involving endocan is ARDS. A previous meta-analysis by Behnoush AH et al. [11] reported a correlation between serum endocan levels and ARDS, and mortality. Considering the comparable pathophysiology related to endothelial dysfunction in OF in both cirrhosis and ARDS, our study showed a similar trend in the association between serum endocan and OF, highlighting its potential as a prognostic marker. Unfortunately, the sample size of patients with respiratory failure in our study was limited (N = 3), which prevented us from establishing a direct association between serum endocan levels and respiratory failure. The previous study also examined the relationship between the severity of ARDS and endocan level, but the results were conflicting. Our findings indicate a correlation between serum endocan levels and OF severity.” (Page 19-20, line 284-294).

**Comment 4**

Mention the clinical utility of your findings for a primary care physician in the discussion.

**Response 4**

Thank you for your valuable feedback. The information has been added to the discussion.

**Revised in the discussion section:**

“This study represents an initial step toward expanding the clinical application of serum endocan. In primary care settings, it can serve as a biomarker for physicians to identify patients with cirrhosis at risk of developing OF. The prospect for future research involves assessing its potential for predicting the development of OF in the high-risk groups because its pathophysiology increases more rapidly than clinical symptoms.” (Page 22, line 334-338).

**Comment 5**

I found some typos and grammatical errors. A native review is required.

**Response 5.**

Thank you for your remarks. The manuscript has been thoroughly reviewed and corrections have been made; additionally, a native review has been completed. The manuscript’s title has been changed to “Association between serum endocan levels and organ failure in hospitalized patients with cirrhosis” (Page 1, line 1-2).

**Reviewer 2**

The authors of the current study aimed to evaluate the association between serum endocan levels and organ failure and 28-day mortality in patients with cirrhosis. The manuscript is written clearly. The results are clearly presented and justify the conclusions.
Minor remarks are as follows:

**Comment 6**

The references for the following statement need to be provided: “The Model for End-stage Liver Disease (MELD) score, Child-Turcotte-Pugh (CTP) score, Chronic Liver Failure Consortium (CLIF-C) ACLF score, and Asian Pacific Association for the Study of the Liver ACLF Research Consortium score were calculated…”

**Response 6**

Thank you for your insightful comment. We have added references for the forementioned scores in the methods section.

**Revised in the methods section:**

“The MELD (Model for End-stage Liver Disease) [20], Child-Turcotte-Pugh (CTP) [21], Chronic Liver Failure Consortium (CLIF-C) ACLF [22], and Asian Pacific Association for the Study of the Liver ACLF Research Consortium (AARC) [23] scores...” (Page 6, line 95-97).

**Comment 7**

The Authors should provide the manufacturer’s name, town and country (in parentheses) for the biochemistry, hematology and coagulation analyzers for the determination of mentioned parameters.

**Response 7**

We thank the reviewer for your comment. The information on the manufacturer’s name, town, and country was added in the methods section.

**Revised in the methods section:**

“Additional laboratories, including biochemistry (Abbott Laboratories, Illinois, USA), hematology (Sysmex, Kobe, Japan), and coagulation (Sysmex, Kobe, Japan), were analyzed according to the manufacturer’s protocol.” (Page 7, line 126-128).

**Comment 8**

The authors are referred to some recent findings of increased serum endocan levels in fatty liver disease and liver fibrosis, also (e.g., doi: 10.2478/jomb-2019-0042)

**Response 8**

We are grateful for the reviewer’s comment. The introduction section has been updated to include information from previous studies on fatty liver disease and liver fibrosis.

**Revised in the introduction section:**

“Similarly, chronic liver disease is associated with endothelial cell dysfunction, and previous studies have indicated that serum endocan levels are correlated with fatty liver disease and advanced fibrosis [17]. In patients with decompensated cirrhosis, serum endocan levels were significantly different from those in the healthy controls [18]. Moreover, serum endocan can serve as an indicator of an unfavorable survival outcome in patients with cirrhosis [19].” (Page 5, line 74-78).

**Reviewer 3**

Wejnaruemarn et al. have performed a study on the association between serum endocan and organ failure by enrolling 116 cirrhotic patients. These are my comments:

**Comment 9**

Calculation of adjusted and unadjusted ORs should be added to the methods section. Variables for which adjustment was performed should also be added.

**Response 9**

Thank you for your insightful comments. The information has been incorporated into the methods and result section.

**Revised in the methods section:**

“including the calculation of adjusted and unadjusted odd ratios,..” (Page 8, line 139).

**Revised in the results section:**

“A univariate analysis was performed to assess possible factors associated with OF. The presence of ascites, serum endocan, IL-6, NLR, creatinine, sodium, TB, and albumin was associated with OFs in cirrhosis. Subsequently, a multivariate logistic regression analysis was performed to examine confounding factors, including significant factors with a *p* < 0.05 identified in the univariate analysis. This included demographic data, such as age, and laboratory data, such as creatinine and TB, to identify factors associated with OF. To address multicollinearity, we removed highly correlated variables. The results indicated that …” (Page 15, line 211-217).

“To assess the prognostic factors in hospitalized patients with cirrhosis, a univariate analysis was carried out, which included factors associated with mortality and indicated..” (Page 17, line 243-244).

**Comment 10**

A comparison should be made with a recent systematic review on the role of endocan in ARDS (doi: 10.1002/hsr2.70044).

**Response 10**

Thank you for your valuable comment. In the discussion section, we have added a comparison with the previous systematic review.

**Revised in the discussion section:**

“In addition to OF in sepsis, another area of extensive research involving endocan is ARDS. A previous meta-analysis by Behnoush AH et al. [11] reported a correlation between serum endocan levels and ARDS, and mortality. Considering the comparable pathophysiology related to endothelial dysfunction in OF in both cirrhosis and ARDS, our study showed a similar trend in the association between serum endocan and OF, highlighting its potential as a prognostic marker. Unfortunately, the sample size of patients with respiratory failure in our study was limited (N = 3), which prevented us from establishing a direct association between serum endocan levels and respiratory failure. The previous study also examined the relationship between the severity of ARDS and endocan level, but the results were conflicting. Our findings indicate a correlation between serum endocan levels and OF severity.” (Page 19-20, line 284-294).

**Comment 11**

A paragraph highlighting the future research perspective and possible gaps in the literature should be added before the conclusion section.

**Response 11**

We are grateful for the reviewer’s comment. The information has been added to the discussion.

**Revised in the discussion section:**

“This study represents an initial step toward expanding the clinical application of serum endocan. In primary care settings, it can serve as a biomarker for physicians to identify patients with cirrhosis at risk of developing OF. The prospect for future research involves assessing its potential for predicting the development of OF in the high-risk groups because its pathophysiology increases more rapidly than clinical symptoms.” (Page 22, line 334-338).

**Comment 12**

The references prior to 2010 could be updated with those after 2010 since they provide more up-to-date findings.

**Response 12**

Thank you for your insightful comments. We have replaced information from the updated references in the introduction, methods, and discussion section.

**Revised in the introduction section:**

“Endocan binds to leukocyte function-associated antigen-1 on the cell surface of lymphocytes, inhibiting its interactions with soluble intercellular adhesion molecule-1 and modulating leukocyte migration to inflammatory sites [9, 10]. Factors such as tumor necrosis factor alpha, interleukin-1, vascular endothelial growth factors C, and lipopolysaccharide from gram-negative bacteria can activate endothelial cells and increase endocan synthesis [9].” (Page 4-5, line 66-70).

**Revised in the methods section:**

“Bacterial infection was diagnosed using standard criteria, including clinical presentation, laboratory results, radiography, and bacterial culture [24-31].” (Page 7, line 118).

**Revised in the discussion section:**

“and its elevated levels on day 1 had a high predictive value for early multiple organ dysfunction in critically ill patients [42]…” (Page 21, line 315-316).

We believe that our responses and manuscript modifications will be satisfactory upon review. Once again, we extend our gratitude to the editors and reviewers for their insightful feedback.

Sincerely,

**Assoc. Prof. Kessarin Thanapirom, M.D., PhD.**

Division of Gastroenterology, Department of Medicine

Chulalongkorn University and King Chulalongkorn Memorial Hospital,

Thai Red Cross Society, Bangkok, Thailand
